# Supplementary material for: Anti-Obesity Effects of the Larval Powder of Steamed and Lyophilized Mature Silkworms in a Newly Designed Adult Mouse Model
Source: Foods. 2023 Sep 28;12(19):3613. doi: 10.3390/foods12193613 (PMC10572763; doi:10.3390/foods12193613)
Supplement: Supplementary file 1 [file foods-12-03613-s001.zip › foods-2631455-supplementary/Table S1.pdf]

Table S1. List of abbreviations (in alphabetical order).

ALP	alkaline phosphatase
ALT	alanine transaminase
ANOVA	one-way analysis of variance
AST	asparagine transaminase
BUN	blood urea nitrogen
BW	body weight
BWG	body weight gain
FER	food efficiency ratio
FI	food intake
HDLC	high-density lipoprotein cholesterol
HFD	high-fat diet
IAT	intra-abdominal adipose tissue
LDLC	low-density lipoprotein cholesterol
MC	methylcellulose
MS	mature silkworm
ND	normal diet
SEM	standard error of the mean
SMSP	steamed and lyophilized mature silkworm powder
SP	silk peptide
TC	total cholesterol
TFI	total food intake
TG	triglyceride
