# Supplementary figures and images for: Anti-Obesity Effects of the Larval Powder of Steamed and Lyophilized Mature Silkworms in a Newly Designed Adult Mouse Model
Source: Foods. 2023 Sep 28;12(19):3613. doi: 10.3390/foods12193613 (PMC10572763; doi:10.3390/foods12193613)

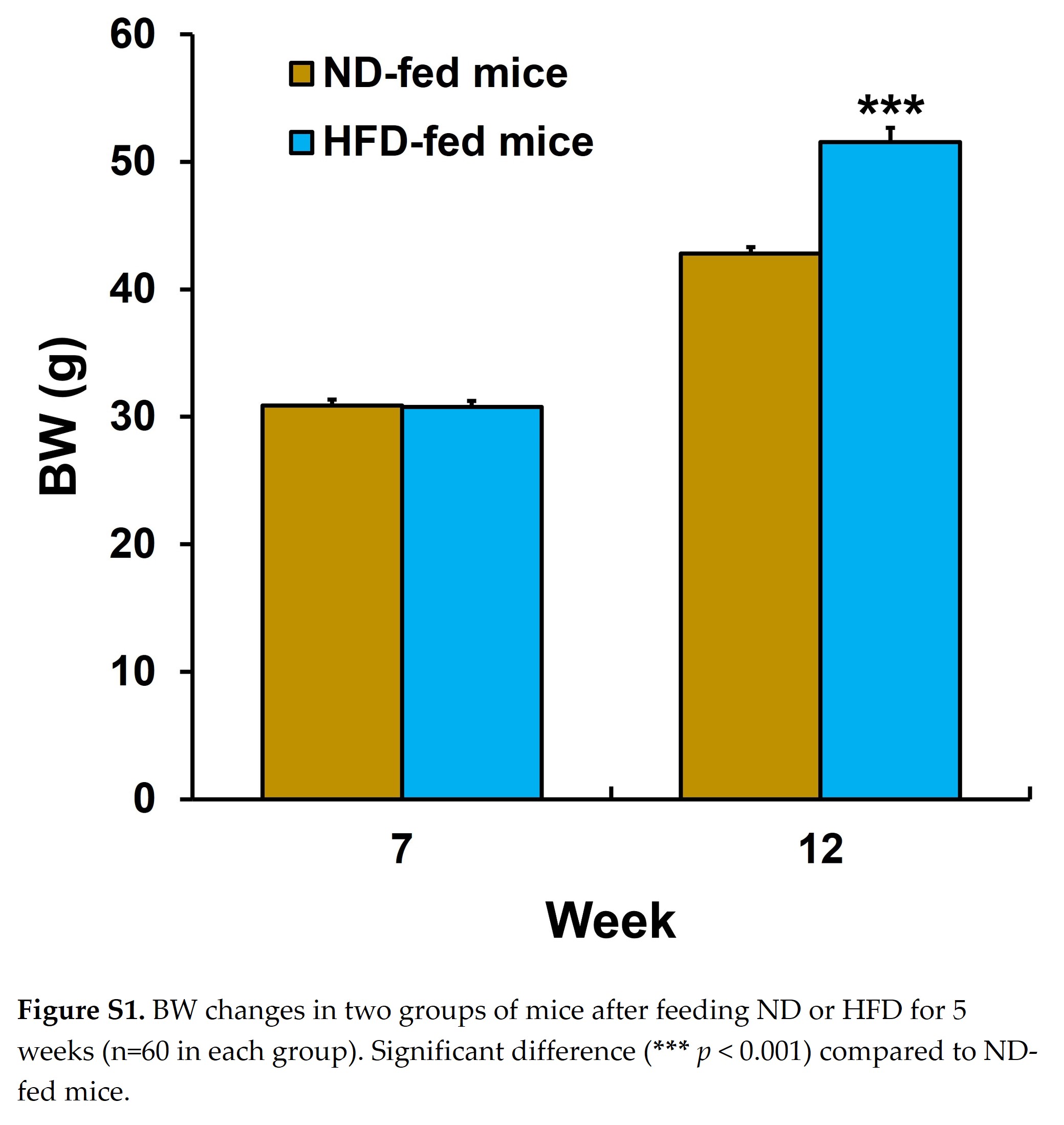

Supplement: Supplementary file 1 [file foods-12-03613-s001.zip › foods-2631455-supplementary/Figure S1.jpg]

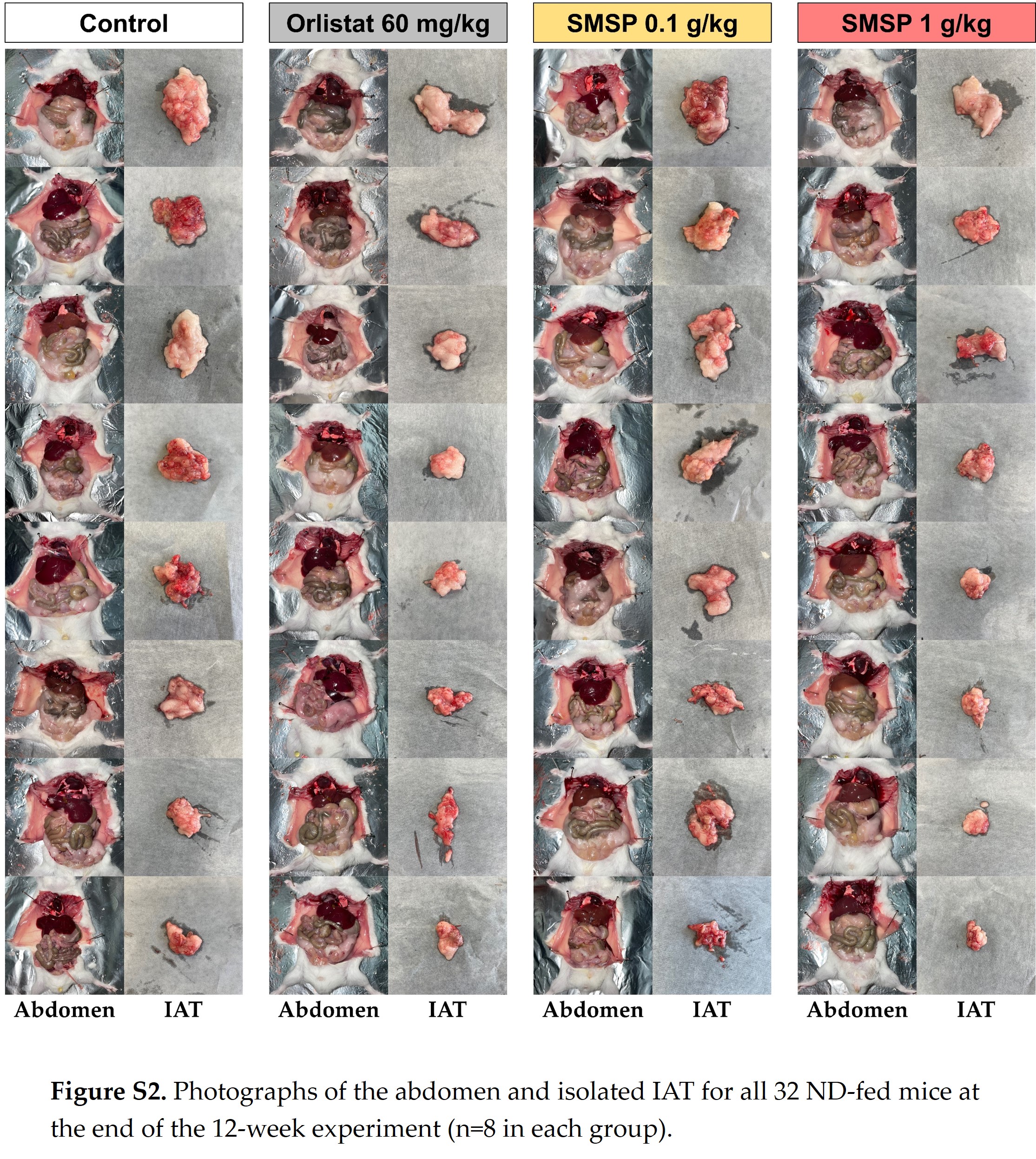

Supplement: Supplementary file 1 [file foods-12-03613-s001.zip › foods-2631455-supplementary/Figure S2.jpg]

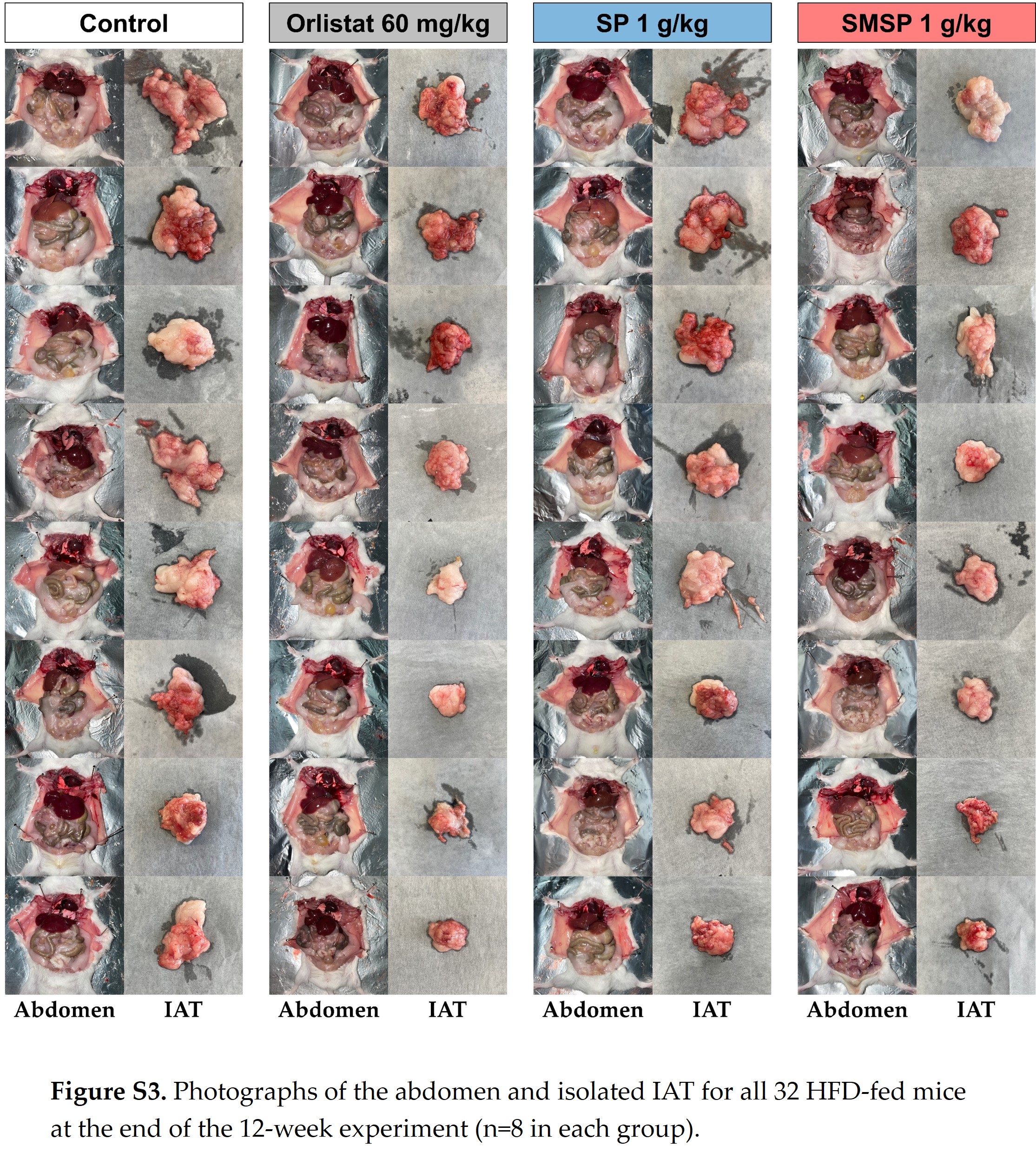

Supplement: Supplementary file 1 [file foods-12-03613-s001.zip › foods-2631455-supplementary/Figure S3.jpg]

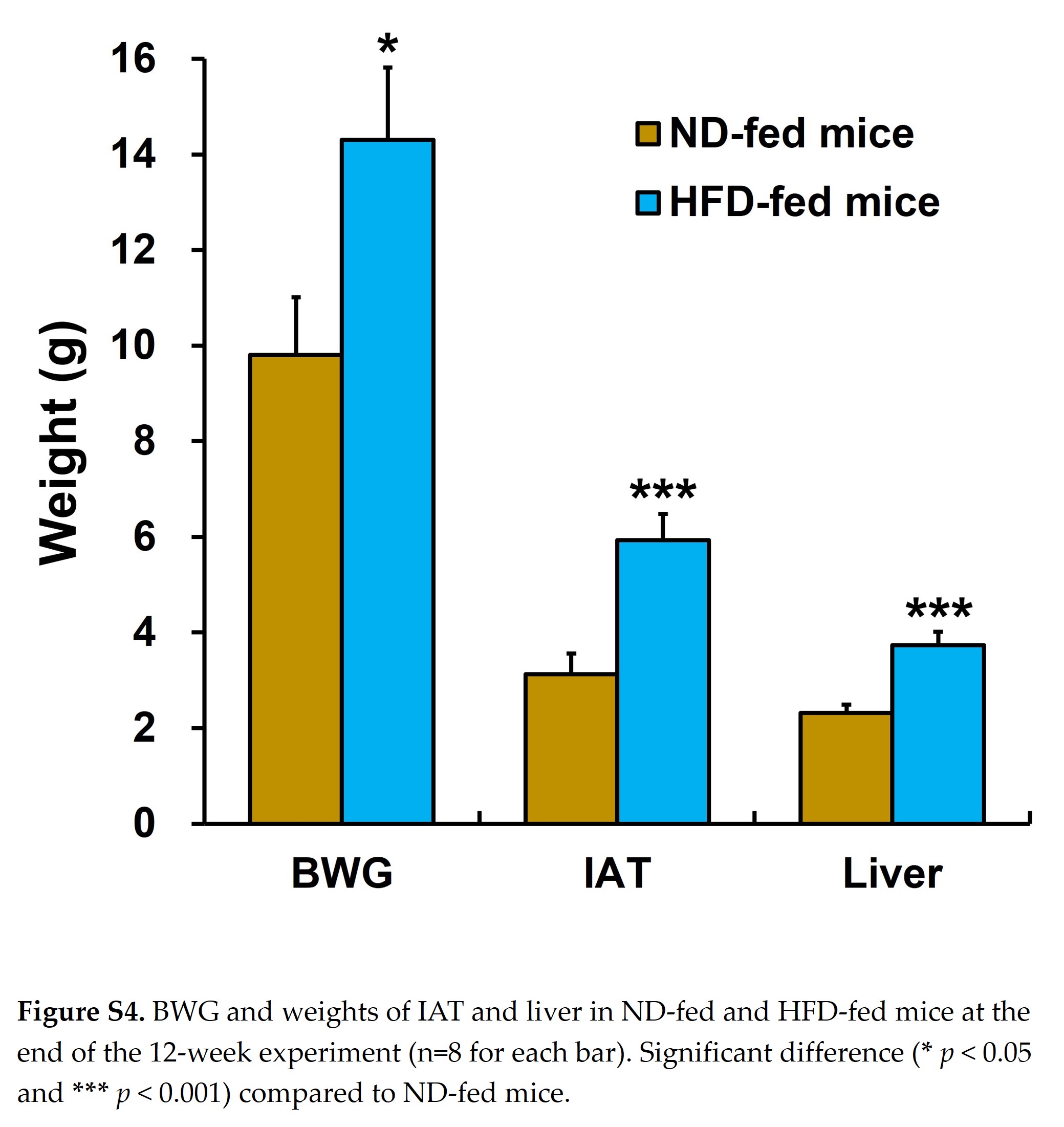

Supplement: Supplementary file 1 [file foods-12-03613-s001.zip › foods-2631455-supplementary/Figure S4.jpg]

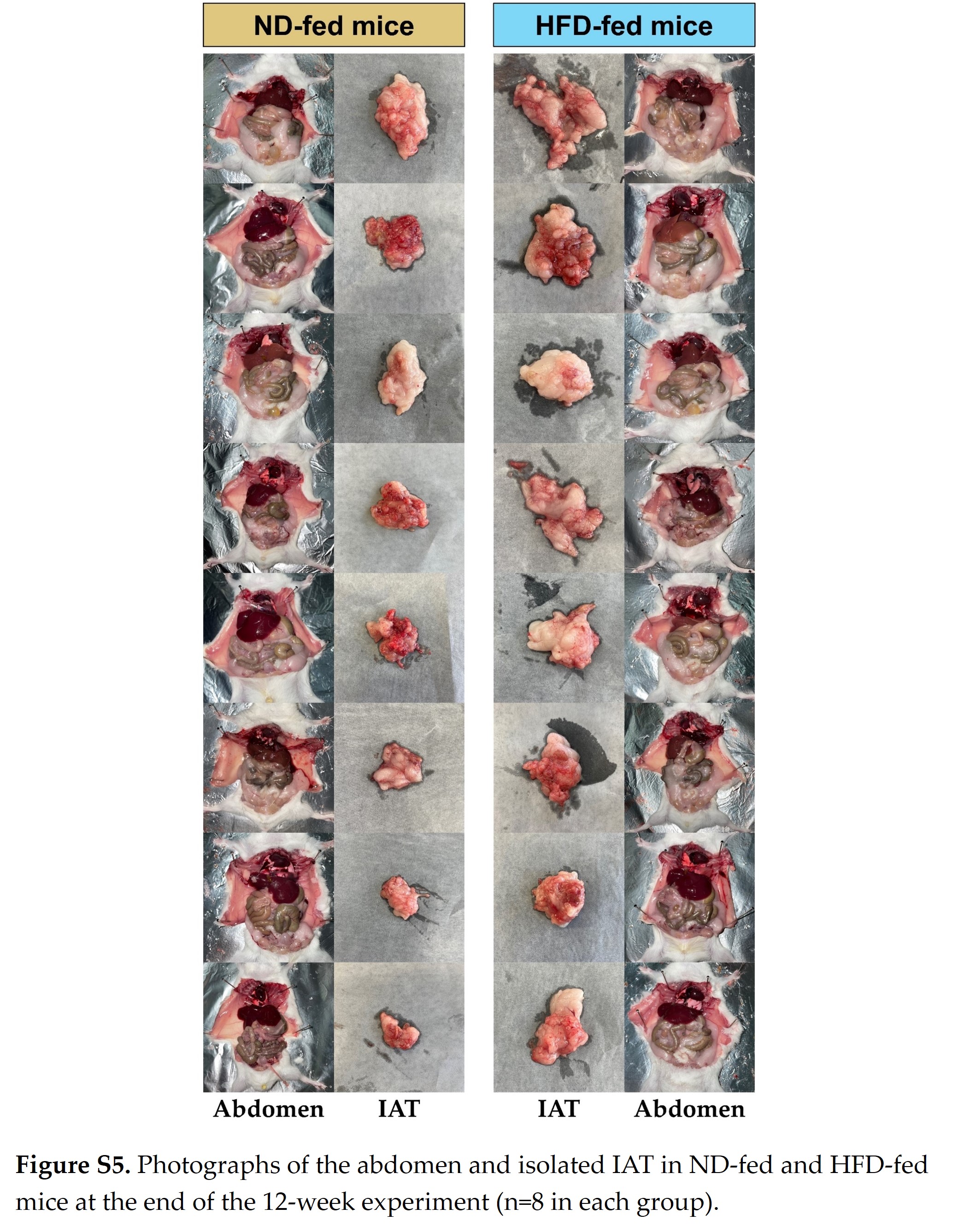

Supplement: Supplementary file 1 [file foods-12-03613-s001.zip › foods-2631455-supplementary/Figure S5.jpg]

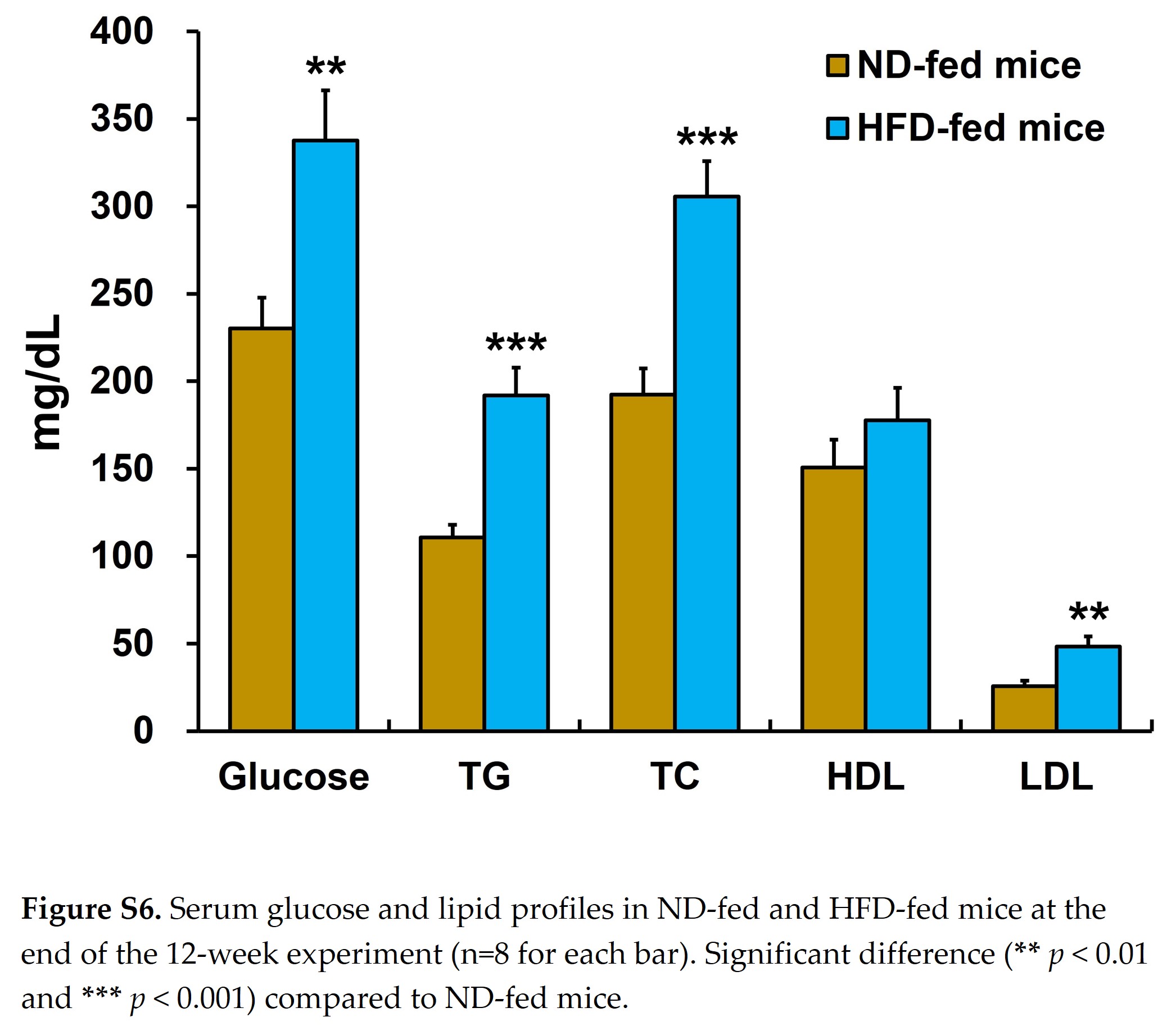

Supplement: Supplementary file 1 [file foods-12-03613-s001.zip › foods-2631455-supplementary/Figure S6.jpg]

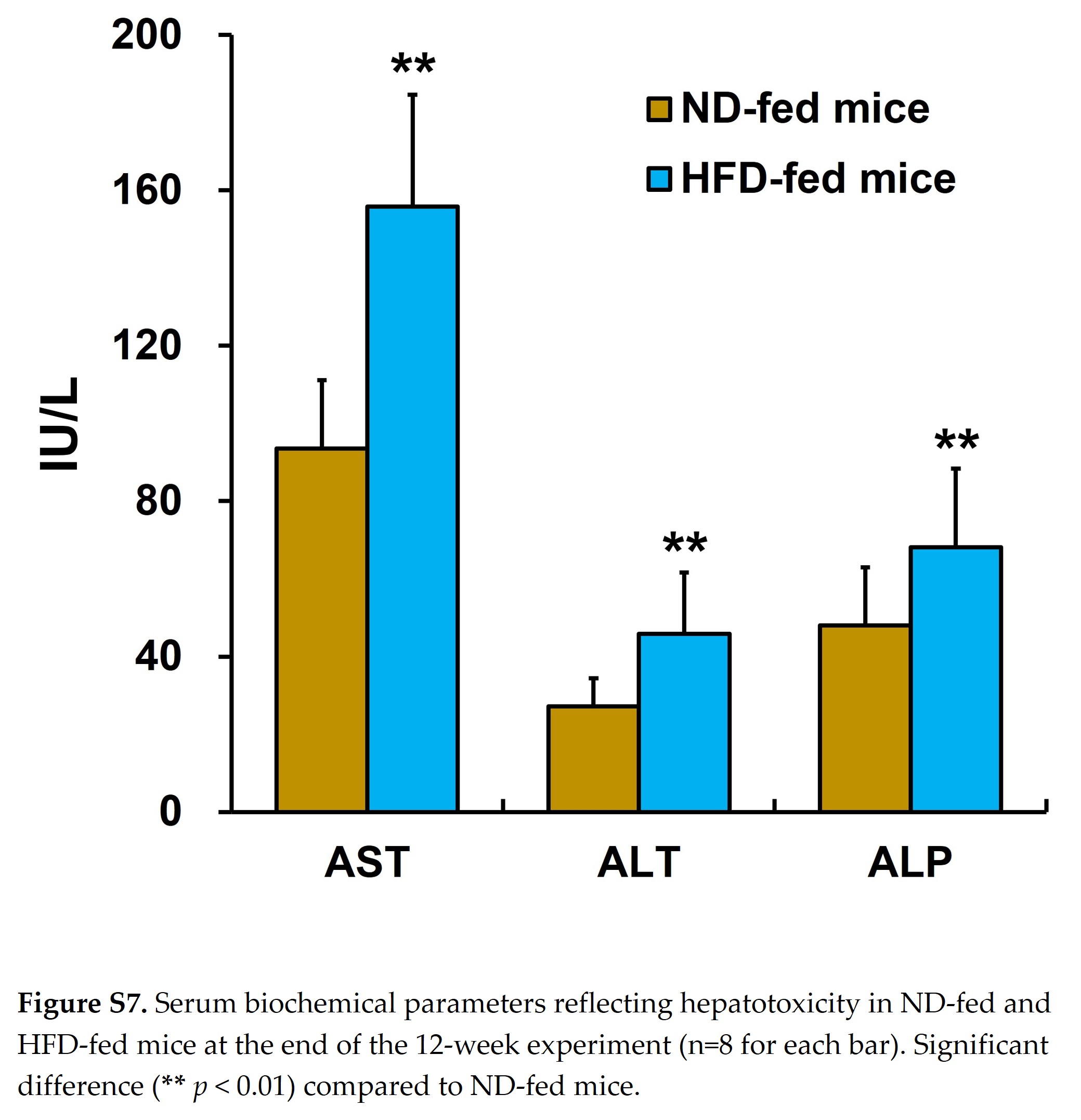

Supplement: Supplementary file 1 [file foods-12-03613-s001.zip › foods-2631455-supplementary/Figure S7.jpg]
